# Alternative carbon sources for the production of plant cellular agriculture: a case study on acetate

**DOI:** 10.3389/fpls.2023.1104751

**Published:** 2023-10-26

**Authors:** Elizabeth C. Hann, Marcus Harland-Dunaway, Adrian J. Garcia, Jonathan E. Meuser, Robert E. Jinkerson

**Affiliations:** ^1^ Center for Industrial Biotechnology, Department of Chemical and Environmental Engineering, University of California, Riverside, Riverside, CA, United States; ^2^ Center for Plant Cell Biology, Department of Botany and Plant Sciences, University of California, Riverside, Riverside, CA, United States; ^3^ Chi Botanic, Alameda, CA, United States

**Keywords:** acetate, carbon source, plant cellular agriculture, plant cell culture, and sucrose

## Abstract

Plant cellular agriculture aims to disrupt the way plant derived products are produced. Plant cell cultures are typically grown with sucrose as the primary carbon and energy source, but alternative carbon sources may have advantages over sucrose including less strain on food systems, lower costs, and more sustainable sourcing. Here we review carbon and energy sources that may serve as alternatives to sucrose in the cultivation of plant cell cultures. We identified acetate as a promising candidate and took the first steps to evaluate its potential for use in growing tobacco plant cell cultures. When added to media containing sucrose, acetate concentrations above 8 mM completely inhibit growth. Lower concentrations of acetate (2-4 mM) can support an increase in dry weight without sucrose but do not provide enough energy for substantial growth. ^13^C labeling indicates that tobacco plant cell cultures can incorporate carbon from exogenous acetate into proteins and carbohydrates. Analysis of transcriptome data showed that genes encoding glyoxylate cycle enzymes are expressed at very low levels compared to genes from the TCA cycle and glycolysis. Adaptive laboratory evolution experiments were able to increase tobacco cell cultures tolerance to acetate, demonstrating the potential for this type of approach going forward. Overall, our results indicate that acetate can be metabolized by plant cell cultures and suggest that further adaptive laboratory evolution or strain engineering efforts may enable acetate to serve as a sole carbon and energy source for tobacco plant cell cultures. This assessment of acetate provides a framework for evaluating other carbon and energy sources for plant cell cultures, efforts that will help reduce the costs and environmental impact, and increase the commercial potential of plant cellular agriculture.

## 1 Introduction

Plant cellular agriculture is the production of plant products from cell culture. Many species of plants have been grown as plant cell cultures to produce a wide variety of medicinal, nutraceutical, food, and biofuel products (Curtin, 1983; Corbin et al., 2016; Yue et al., 2016; Norouzi et al., 2022). Production of plant-derived products through plant cell cultures can have many benefits over production from a traditional, whole plant. Over 30 target compounds have been produced in plant cell cultures at levels exceeding the content in traditionally grown plants (Gaosheng and Jingming, 2012). Perhaps the most well-known example is the production of the anti-cancer drug Paclitaxel (e.g. Taxol), a compound found at low levels in the bark of the Pacific yew tree (*Taxus brevifolia*) (Camidge, 2001). Only through plant cell cultures could sufficient quantities of the drug be made, achieved through fermentations at the 75,000 liter scale (Expósito et al., 2009).

### 1.1 Sucrose is the most utilized carbon and energy source for plant cell cultures

**Figure 1:**
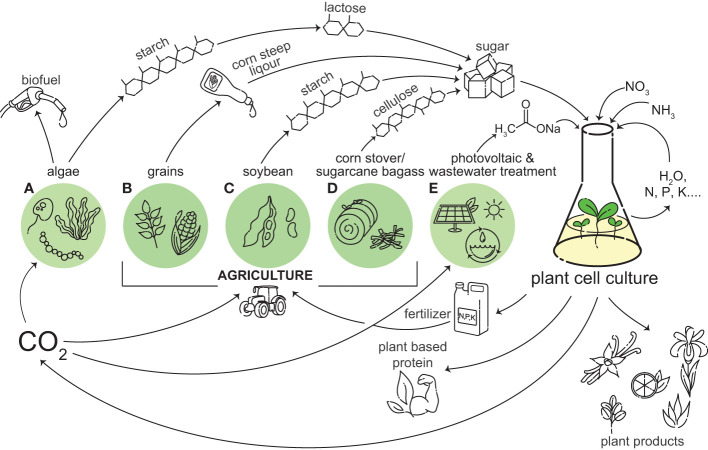
Plant cell culture can be integrated into a circular bio-economy. A wide variety of plant based products and important chemicals can be produced from plant cell cultures, including vanilla, citrus, Iris, aloe, and rubber. Plant cell culture can grow on outputs from agriculture and photovoltaics and wastewater treatment, and in turn plant cell culture can provide inputs into these systems, including CO_2_ and fertilizer. Sugar, like sucrose and glucose, are currently used as carbon and energy sources for plant cell culture growth. Beyond traditional sources, these sugars can be obtained from the by-products of algae **(A)**, grains **(B)**, soybean **(C)**, and corn stover/sugarcane bagasse **(D)**. We are proposing acetate as a future alternative energy source for plant cell culture. Some sources of acetate include photovoltaic powered electrochemical catalysis and wastewater treatment **(E)**. Plant cell culture produces CO_2_ through respiration, which can be used for the photosynthetic growth of algae and crops **(A–D)**, and in photovoltaic powered electrochemical catalysis **(E)**. If plant cell culture can utilize a carbon source derived from something it is providing inputs to, it can create a closed-loop system.

Plant cell cultures are most often grown heterotrophically, and thus require a carbon and energy source. Sucrose from sugar cane or beets is the most common carbon source for plant cell cultures. Plant cells have evolved a suite of transporters and enzymes to utilize sucrose as a carbon source (Chen et al., 2015). Despite the native ability of plant cell cultures to utilize sucrose, there are several drawbacks to sucrose as a carbon source. It is a staple food consumed globally, so using it for the production of plant cell cultures directly competes with the food supply. As plant cellular agriculture is expanded, increasing sucrose consumption by the industry may prompt ethical questions and consumer pushback due to the diversion of food for the production of non-edible products, similar to what was seen in the ‘food versus fuel’ debate in first generation biofuels (Tomei and Helliwell, 2016). There are also sustainability issues surrounding the production of sucrose. The farming of 1 kg of sucrose from sugarcane releases up to 5 kg of CO_2_ equivalents. While 1 kg of sucrose from beet sugar releases less, on average 1.8 kg of CO_2_ equivalents, both crops require about 2 square meters of farmland per year per kilogram produced (Poore and Nemecek, 2018). Sucrose has sufficed for the cultivation of plant cell cultures to date, but as interest in plant cell culture increases, there is a need to investigate alternative carbon and energy sources which may be more abundant, less expensive, and more sustainable (**Figure 1**).

### 1.2 Alternative carbon and energy sources for plant cell culture

Agricultural waste streams that produce carbohydrates, starch, and cellulose could be utilized as alternative sources of carbon for the cultivation of plant cell cultures. Many plant-based industries generate large quantities of carbohydrates as co-products. It has previously been reported that plant cell cultures can utilize a variety of mono- and di- saccharides, with glucose, sucrose, and maltose being the best substrates for growth (Fowler, 1982). Corn steep liquor, which is produced by the corn refining industry, contains 10-20% carbohydrates (mono-, di- saccharides, lactose) and 40% nitrogen (protein, peptides, amino acids) as well as abundant vitamins, minerals, and positive growth factors (Hull et al., 1996; Li et al., 2016). Waste streams that contain starches could also be useful for the cultivation of plant cell cultures. Some plant cell cultures have been grown with starch as a substrate, but at rates and conversion levels much lower than with glucose (Fowler, 1982). In the actively growing plant-based protein market, proteins are extracted from legumes, leaving behind approximately 40% to 60% of the original weight as starch, which could be repurposed for the cultivation of plant cell cultures. Agricultural residues like corn stover, sugarcane bagasse, and wood wastes are composed of cellulose, hemicellulose, and lignin. Lignocellulosic materials have been proposed as feedstocks for the production of several bioproducts, most notably cellulosic biofuels, but this material could also serve as a plant cell culture carbon source. Cellulose cannot be directly digested by plant cell cultures, however enzymatic or chemical hydrolysis can free sugars from cellulose to then be used in the cultivation of plant cell cultures.

Algae could also serve as a carbon source for the cultivation of plant cell cultures. Algae are grown for aquaculture feed, nutraceuticals (Spolaore et al., 2006), and biofuels (Radakovits et al., 2010). Algae produce carbohydrates that could be used as carbon for plant cell culture cultivation, such as starch, glucose, maltose, and other sugars. Algal monosaccharides and disaccharides can most likely be directly utilized by plant cell cultures. Starches can be enzymatically saccharified into glucose which could then be used to cultivate plant cell cultures (Lee and Lee, 1996; Lee et al, 1999). Integrating the manufacture of high-value products from plant cell cultures into a traditional farm, or a production facility for plant-based food or biofuels, could improve upon profitability, reduce waste, and generate valuable co-products by making use of compounds otherwise wasted.

### 1.3 Acetate as a potential carbon and energy source for plant cell culture

Acetate could serve as an alternative carbon source for plant cell cultures. It is abundant and produced by a variety of methods. Most acetate is currently produced through chemical synthesis from petroleum-derived feedstocks, however there is a small amount of acetate produced through biological fermentation (Kiefer et al., 2021). Some alternative methods for acetate production include hydrolysates of lignocellulosic biomass, electrochemical catalysis, and microbes. Hydrolysates of lignocellulosic biomass, an abundant and renewable resource, can contain up to 17.2 g l^-1^ acetate (Gong et al., 2016). Microbes can be used to produce acetate through fermentation of C1 gasses (Kantzow et al, 2015) and electrosynthesis (Nevin et al., 2011). Electrochemical catalysis can produce acetate from carbon dioxide and electricity (Hann et al., 2022; Overa et al., 2022). Although these alternative methods of acetate production have been largely unproven at scale, with additional research and development, acetate may be produced with less environmental impact than sucrose.

Improvements to alternative methods of acetate production may lead to a product that can compete with sucrose on an economic level as well. At the time of writing, sucrose costs $0.417 kg^-1^ and conventionally produced acetate costs $0.58 kg^-1^. Sucrose and acetate have very similar energy content on a per mass basis, 16.5 and 14.6 kJ g^-1^ respectively. If the cost of acetate can be brought down by even 30%, it could out-compete sucrose economically. CO_2_ electroreduction can produce acetate at less than $0.5 kg^-1^ using electricity below $0.03 kWh^-1^ (Jouny et al, 2019), a target set to be reached by 2030 with photovoltaic technology (Solar Energy Technologies Office Updated 2030 Goals for Utility-Scale Photovoltaics; Crandall et al., 2023). With minimal improvements, this process could replace or compete with current petroleum-reliant processes as an environmentally friendly alternative acetate production method, and the acetate produced could compete with sucrose as a carbon and energy source to support an alternative bioeconomy.

The physical, chemical, and biological properties of acetate make it a desirable alternative carbon and energy source for plant cell cultures. Acetate is miscible in water to high concentrations, which is ideal for the heterotrophic growth of organisms in a liquid culture. Unlike sucrose, acetate can diffuse across the cell membrane of many organisms without the need of specific transporters. Acetic acid can be used to buffer media pH, and because acetate consumption alters media pH, pH measurements can be used as an additional on-line process parameter to monitor growth of the culture. (Kiefer et al., 2021).

Acetate is an established carbon and energy source for many microorganisms and has already been incorporated into a variety of fermentation processes. This includes processes using bacteria, such as *E. coli* and *C. necator*, to produce biodegradable and biocompatible thermoplastics (Dai et al., 2007; Garcia-Gonzalez and De Wever, 2018; Sun et al., 2020). Oleaginous yeasts have been grown with acetate as the sole carbon and energy source to produce lipids (Béligon et al., 2015; Huang et al., 2016; Xu et al., 2017; Zhang et al., 2019). Photosynthetic purple bacteria have been cultivated with acetate for hydrogen gas production (Barbosa et al., 2001; Oh et al., 2004; Fang et al, 2005). *Chlamydomonas reinhardtii*, a green algae and model organism closely related to vascular plants, is grown primarily on acetate in laboratory settings (Harris et al, 1989). Recent, comprehensive literature reviews describe the microbial production of biochemicals from acetate, but plant cell culture is not included (Lim et al., 2018; Kutscha and Pflügl, 2020; Kiefer et al., 2021).

**Figure 2:**
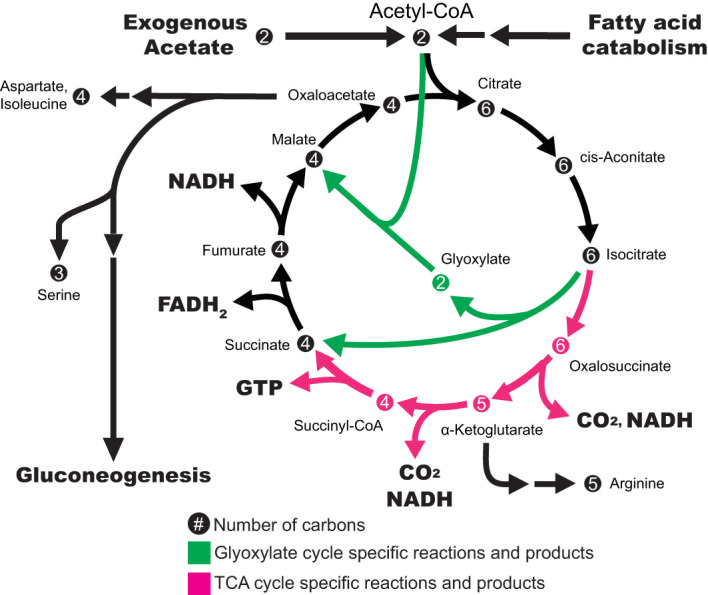
Model of exogenous acetate consumption in plant cells and how the resulting acetyl-CoA is metabolized through the TCA or glyoxylate cycle. The TCA cycle and the glyoxylate cycle are depicted showing their distinct steps in pink and green respectively, with steps in black shared between both cycles. The TCA cycle converts acetyl-CoA into energy in the form of NADH, GTP, and FADH_2_, however there are two carbons lost in the TCA cycle which limits anabolic metabolism. The glyoxylate cycle does not produce as much energy but it skips the two decarboxylation reactions in the TCA cycle, which allows cells to upgrade the two carbon acetyl-CoA molecules into large carbon backbones that can be used to produce proteins or can be converted into sugars through gluconeogenesis. Both fatty acid catabolism and exogenously supplied acetate can be metabolized in the same way to provide energy and build cell biomass. Numbers within circles indicate the number of carbons within that molecule.

Vascular plants natively metabolize acetate via the glyoxylate cycle during early postgerminative growth. Fatty acids are released from storage lipids in the endosperm and catabolized through beta-oxidation to make the bio-active form of acetate, acetyl-CoA. The glyoxylate cycle bypasses the two steps in the tricarboxylic acid (TCA) cycle in which a CO_2_ molecule is lost, enabling plants to build two carbon acetyl-CoAs into larger molecules which serve as the precursors for gluconeogenesis, which produces glucose (**Figure 2**). This process converts lipids into acetate and then into sugar (Buchanan et al, 2015). Storage lipids, by way of acetate, are the sole carbon and energy source to sustain growth for the short period of time in plant development before photosynthesis begins and becomes the primary source of carbon and energy.

Acetate has been demonstrated as a suitable carbon and energy source for a variety of bioprocesses, however, acetate has not been widely explored as a carbon and energy source for the heterotrophic cultivation of plants. Related published literature focuses primarily on lipid metabolism, and observations about utilization of acetate as an energy source are incidental. The largest collection of existing literature in which exogenous acetate is added to the growth media of plant cell cultures is for isotopic or radioactive labeling experiments where acetate is fed to plant cell cultures, usually in addition to sucrose, primarily to investigate fatty acid biosynthesis (**Table 1**).

**Table 1:** Previous studies that investigate acetate incorporation into plant cell culture metabolites.

Publication	Plant cell culture species	Labeled acetate used	Metabolites examined
Fletcher and Beevers, 1970	Paul’s scarlet rose	1-^14^C	lipids, CO_2_, organic acids, amino acids, TCA cycle intermediates, and protein
Turnham and Northcote, 1984	Oil palm (*Elaeis guineensis*)	1-^14^C	lipids
Ashworth et al, 1987	Maize *(Zea mays)*	1-^13^C and 2-^13^C	lipids, TCA cycle intermediates, amino acids
Chappell et al., 1989	Tobacco (*Nicotiana tabacum*)	^14^C	isoprenoids
Shintani and Ohlrogge, 1995	Tobacco (*Nicotiana tabacum*)	1-^14^C	lipids
Lee and Lee, 1996	Rice (*Oryza sativa*)	1-^14^C	none (rate of uptake)
Marty et al., 1997	*Datura stramonium* and *Nicotiana plumbaginifolia*	2-^13^C	amino acids, TCA cycle intermediates
Lee et al, 1999	Carrot (*Daucus carota*)	1-^14^C	none (rate of uptake)
Hoang and Chapman, 2002	Tobacco (*Nicotiana tabacum*)	2-^14^C	lipids
Andre et al., 2012	*Brassica napus*	1-^14^C and 2-^14^C	lipids
Keereetaweep et al., 2018	*Arabidopsis thaliana*	1-^14^C	lipids

Some previous studies suggest that there may be potential for acetate to serve as a primary carbon and energy source for plants, although there is no convincing published evidence that plants can grow on acetate alone. Lee and Lee, 1996 grew rice (*Oryza sativa*) plant cell cultures with 10 mM potassium acetate alone in the dark, however these cells reached less than 40% of the increase of fresh weight seen in cells grown with glucose (10 mM). Additionally, cells grown with acetate reached stationary phase at day two of the six-day growth curve, compared to cells grown with glucose which did not reach stationary phase until day five (Lee and Lee, 1996). Previous studies have also suggested that acetate may negatively affect growth of plant cell cultures. Rice (*Oryza sativa*) and carrot (*Daucus carota*) plant cell cultures have been reported to grow in 10 mM acetate without mention of growth inhibition (Lee and Lee, 1996; Lee et al, 1999), while concentrations above 0.2 mM supplemental acetate were said to cause cell death to cucumber (*Cucumis sativus*) plant cell cultures (Graham et al, 1994) (**Table 2**). These inconsistencies in the literature make it difficult to conclude if acetate could serve as a viable carbon and energy source for plant cell cultures.

**Table 2:** Growth of plants with exogenous acetate.

Publication	Species	Growth type	Acetate: supplemental or sole carbon source	Acetate type	Concentration	Notes
Ashworth et al., 1985	Maize *(Zea mays)*	Plant cell culture	Supplemental	sodium, potassium, and ammonium acetate	0.5-10 mM	A range of 0-10 mM tested, 2.5 and higher affected final packed cell volume.
Graham et al, 1994	Cucumber (*Cucumis sativus*)	Plant cell culture	Supplemental	not specified	0.2 mM	Higher concentrations of acetate were toxic and resulted in rapid cell death.
Lee and Lee, 1996	Rice (*Oryza sativa*)	Plant cell culture	Both	potassium acetate	10 mM	Cells grown with acetate and/or glucose.
Marty et al., 1997	*Datura stramonium* and *Nicotiana plumbaginifolia*	Plant cell culture	Supplemental	sodium acetate	4 mM	Dry weight at 20 days was not affected when 4 mM acetate was added after 5 days of growth.
Lee et al, 1999	Carrot (*Daucus carota*)	Plant cell culture	Supplemental	potassium acetate	10 mM	Cells grown with acetate and glucose.
Turner et al., 2005	*Arabidopsis thaliana*	Vascular	Supplemental	sodium acetate	3.5 mM	No effects to plants seen at 1 and 2 mM, at 3.5 mM growth decreased 30-40%.
Lim et al, 2018	*Arabidopsis thaliana*	Vascular	Sole	sodium acetate	1 mM	Plants grew on acetate alone as shown by fresh weight.
Fu et al., 2020	*Arabidopsis thaliana*	Vascular	Supplemental	sodium acetate	1 mM	Shoot and root growth inhibited.
Hann et al., 2022	Lettuce (*Lactuca sativa*)	Vascular	Sole	acetic acid	1-10 mM	Growth inhibited at 1 mM concentrations and higher.

In this work, we investigated the use of acetate for cultivation of tobacco plant cell cultures. We measured how acetate affects growth and grew tobacco plant cell cultures with acetate as the sole carbon and energy source. Metabolomics analysis revealed how acetate is metabolized in these cells and analysis of transcriptome data revealed the expression levels of key enzymes in acetate metabolism. Finally, adaptive laboratory evolution experiments were conducted to increase acetate tolerance of the plant cell cultures.

## 2 Results

### 2.1 Evaluation of acetate as an alternative carbon source for tobacco plant cell culture

**Figure 3:**
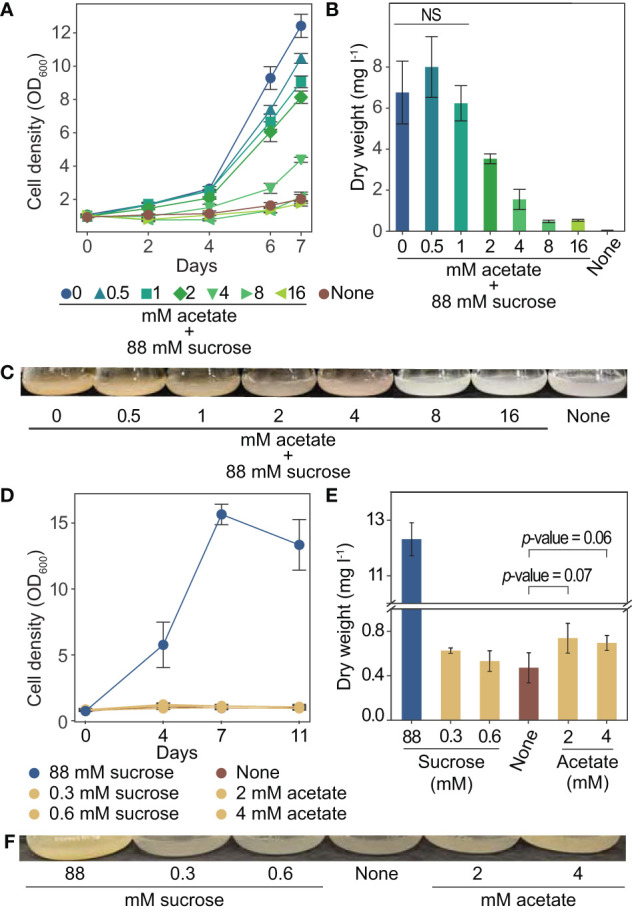
Growth of tobacco plant cell culture is inhibited by exogenous acetate above 8 mM. Tolerable concentrations of acetate serving as the sole carbon and energy source support an increase in dry weight. **(A-C)** Tobacco plant cell cultures were grown in media containing sucrose and the addition of 0 to 16 mM acetate. Negative control “none” was grown in media with no primary carbon or energy source (no sucrose or acetate). Each data point is representative of four replicates. Error bars represent standard deviation. **(A)** Cell density measurements (OD_600_) over one week of growth. **(B)** Dry weight biomass measurements taken after one week of growth. A two-tailed unpaired t-test was used to compare growth in experimental media to growth in media containing sucrose and 0 mM acetate, *p*-values are: 0.288 for 0.5 mM acetate, 0.569 for 1 mM acetate, 0.006 for 2 mM acetate, 0.0006 for 4 mM acetate, 0.0002 for 8 mM and 16 mM. NS denotes no significant difference from 0 mM acetate 88 mM sucrose (*p*-value > 0.05) **(C)** Images of flasks were taken after one week of growth, images are representative of all replicates. **(D–F)** Tobacco plant cell cultures were grown in media containing 2 mM or 4 mM acetate (1.8 kJ l^-1^ and 3.7 kJ l^-1^) (no sucrose). Additional cultures were also grown in media containing the energetic equivalent amount of sucrose (0.33 mM and 0.66 mM, 1.8 kJ l^-1^ and 3.7 kJ l^-1^) (no acetate). Cells were grown in media containing sucrose (495 kJ l^-1^ approximately 88 mM) (no acetate) as a positive control. Negative control “none” was grown in media with no primary carbon or energy source (no sucrose or acetate). Each data point is representative of three replicates. Error bars represent standard deviation. **(D)** OD_600_ of cultures over eleven days of growth. **(E)** Dry weight biomass measurements taken after two weeks of growth. A two-tailed unpaired t-test was used to compare growth in experimental media to growth in media containing no primary carbon or energy source (“none”), *p*-values are: 0.12 for 0.3 mM sucrose, 0.56 for 0.6 mM sucrose, 0.07 for 2 mM acetate, 0.06 for 4 mM acetate. NS denotes no significant difference from “none” (*p*-value > 0.05). **(F)** Images of flasks taken after two weeks of growth, images are representative of all replicates.

We used tobacco (*Nicotiana tabacum*) BY-2 plant cell cultures for our investigation into acetate as an alternative carbon source for plant cell culture. These cells have a fast doubling time and are commonly used for a range of studies (Nagata et al, 2004). To better understand the relationship between acetate and tobacco plant cell cultures, we grew cells for one week in media containing up to 16 mM acetate in addition to the standard amount of sucrose typically used in tobacco plant cell culture medium (approximately 88 mM) (Murashige and Skoog, 1962). We found that the addition of acetate above 1 mM resulted in decreased growth rates and significantly less biomass after one week of growth, with 2 mM acetate reducing the final dry weight by almost 50% (**Figures 3A–C**).

To determine if acetate can support growth on its own, we grew tobacco plant cell cultures with acetate in place of sucrose for two weeks. We used the highest levels of acetate that were found to not completely prohibit growth (2 and 4 mM). As a control, we also grew cultures with sucrose as the only energy source, in concentrations to match the energetic equivalent of 2 and 4 mM acetate. The energetic equivalent was determined using the enthalpy of combustion of acetate (14.58 kJ g^-1^) and sucrose (16.5 kJ g^-1^). Media made with 2 and 4 mM acetate has about 1.8 and 3.7 kJ l^-1^, which is energetically equivalent to about 0.3 and 0.6 mM sucrose. The energy content of standard tobacco plant cell culture medium is approximately 495 kJ l^-1^ from sucrose (Murashige and Skoog, 1962). The growth observed in media with 1.8 and 3.7 kJ l^-1^, from sucrose or acetate, was much less than in the standard media (**Figure 3D**). However, slight increases in biomass were observed for cultures grown with 2 and 4 mM acetate over cultures grown with no sucrose or acetate (**Figure 3E**, *p*-values 0.07 and 0.06 respectively as determined by a two-tailed t-test).

### 2.2 Metabolism of exogenous acetate

**Figure 4:**
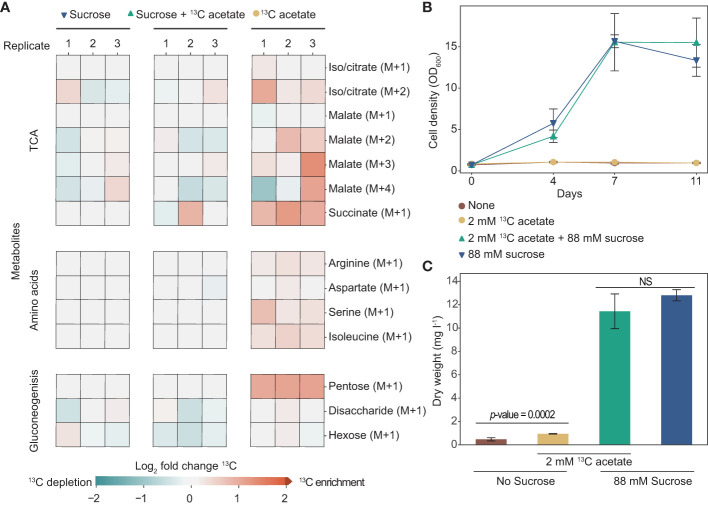
Tobacco cell culture can incorporate exogenous acetate into metabolites involved in the tricarboxylic acid cycle (TCA), gluconeogenesis, and amino acids. **(A)** Heat map showing log_2_-fold enrichment of ^13^C in metabolites of tobacco plant cell cultures grown in media containing sucrose, sucrose plus 2 mM [2-^13^C] acetate, or with just 2 mM [2-^13^C] acetate (no sucrose). Analysis was done on cells after two weeks of growth. Each column represents one biological replicate. Citrate and isocitrate were indistinguishable under our metabolomics analysis methodology. **(B)** OD_600_ of cultures during eleven days of growth. **(C)** Dry weight biomass measurements taken after two weeks of growth. A two-tailed unpaired t-test was used to compare growth of cells in media with sucrose with and without the addition of 2 mM [2-^13^C] acetate, the *p*-value is 0.20. NS denotes no significant difference between cells grown in media containing sucrose (*p*-value > 0.05). A two-tailed unpaired t-test was used to compare growth of cells in media containing no sucrose, with and without the addition of 2 mM [2-^13^C] acetate, the *p*-value is 0.0002. **(B, C)** Each data point is representative of three replicates. Error bars represent standard deviation.

To better understand acetate metabolism in tobacco plant cell cultures, we tracked its incorporation using heavy isotope [2-^13^C] acetate. We grew cells for two weeks in media with either 88 mM sucrose, 2 mM [2-^13^C] acetate, or both and used LC-MS to identify ^13^C incorporation into a variety of metabolites (**Figure 4A**). Tobacco plant cell cultures grown with [2-^13^C] acetate have ^13^C enrichment in metabolites involved in the TCA cycle, gluconeogenesis, and amino acid biosynthesis. Interestingly, we found that cells grown with [2-^13^C] acetate without sucrose have much more substantial ^13^C enrichment than cells grown with sucrose plus [2-^13^C] acetate. For example, ^13^C-labeling of pentose in cells grown with just [2-^13^C] acetate is two-fold higher than in cells grown with sucrose plus [2-^13^C] acetate. Notably, cells grown in media with sucrose plus [2-^13^C] acetate grew considerably more than cells grown in media with just [2-^13^C] acetate as a carbon source (no sucrose), ending the two-week-long experiment with dry weights of over 11 mg l^-1^ and less than 1 mg l^-1^ respectively (**Figures 4B–C**).

### 2.3 Gene expression of enzymes involved in acetate metabolism

**Figure 5:**
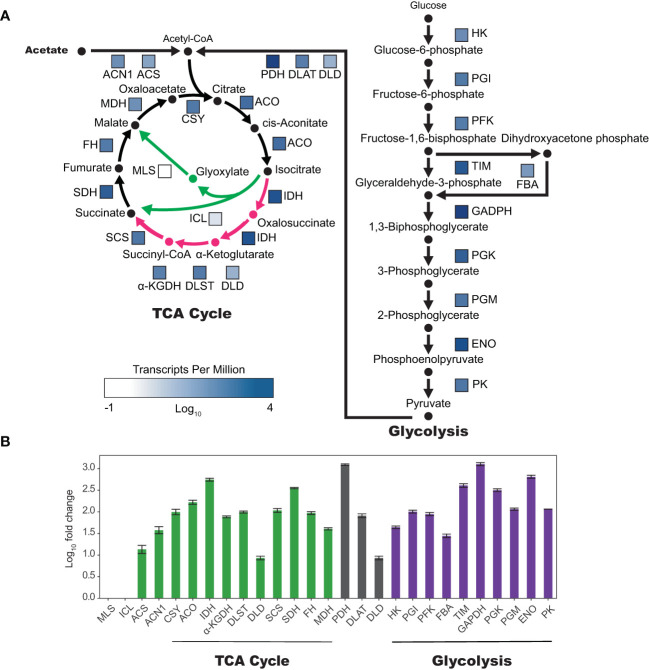
Genes unique to the glyoxylate cycle are expressed in very low levels in tobacco BY-2 cells. **(A)** Colored boxes next to enzyme abbreviations correspond to relative expression as log_10_(TPM)(transcripts per million). TCA and glyoxylate cycles are represented on the left, with TCA cycle specific reactions and products in pink and glyoxylate cycle specific reactions and products in green. **(B)** Log_10_ of fold change compared to TPM of isocitrate lyase (ICL). Two biological replicates, error bars represent standard deviation. α-KGDH, DLST, and DLD make up the alpha-ketoglutarate dehydrogenase complex (α-KGDHC). PDH, DLAT, and DLD make up the pyruvate dehydrogenase complex (PDHC). Abbreviations for enzyme names are as follows: malate synthase (MLS), isocitrate lyase (ICL), acetyl-CoA synthetase (ACS), acetate non-utilizing 1 (ACN1), citrate synthase (CSY), aconitase (ACO), isocitrate dehydrogenase (IDH), α-ketoglutarate dehydrogenase (α-KGDH), dihydrolipoamide succinyltransferase (DLST), dihydrolipoamide dehydrogenase (DLD), succinyl-coa ligase (SCS), succinate dehydrogenase (SDH), fumarase (FH), malate dehydrogenase (MDH), pyruvate dehydrogenase (PDH), dihydrolipoamide acetyltransferase (DLAT), dihydrolipoyl dehydrogenase (DLD), hexokinase (HK), phosphoglucose isomerase (PGI), phosphofructokinase (PFK), fructose-bisphosphate aldolase (FBA), triosephosphate isomerase (TIM), glyceraldehyde-3-P dehydrogenase (GADPH), phosphoglycerate kinase (PGK), phosphoglyceromutase (PGM), enolase (ENO), pyruvate kinase (PK).

To better understand the transcriptional regulation of acetate metabolism in tobacco plant cell cultures, we examined the gene expression of enzymes involved in the TCA cycle, glyoxylate cycle, and gluconeogenesis (**Figure 5**). Analysis of BY-2 plant cell culture RNA-seq data (Yang et al., 2015; Yang et al., 2017) revealed high expression levels of enzymes in both the TCA cycle and gluconeogenesis, with average transcripts per million (TPM) for enzymes in the TCA cycle being 368 and glycolysis 717. However enzymes specific to the glyoxylate cycle, malate synthase (MLS) and isocitrate lyase (ICL), were expressed at low levels, with average TPMs of 0.43 and 2.29 respectively. The bifurcation point between the TCA cycle and the glyoxylate cycle is at isocitrate, which can enter the TCA cycle via isocitrate dehydrogenase (IDH) or the glyoxylate cycle via ICL. Interestingly, IDH is expressed higher than any other TCA cycle enzyme, at 1,270 TPM, which is 556-fold higher than ICL (**Supplemental Table 1**).

### 2.4 Adaptive laboratory evolution experiments to improve acetate tolerance in tobacco plant cell culture

**Figure 6:**
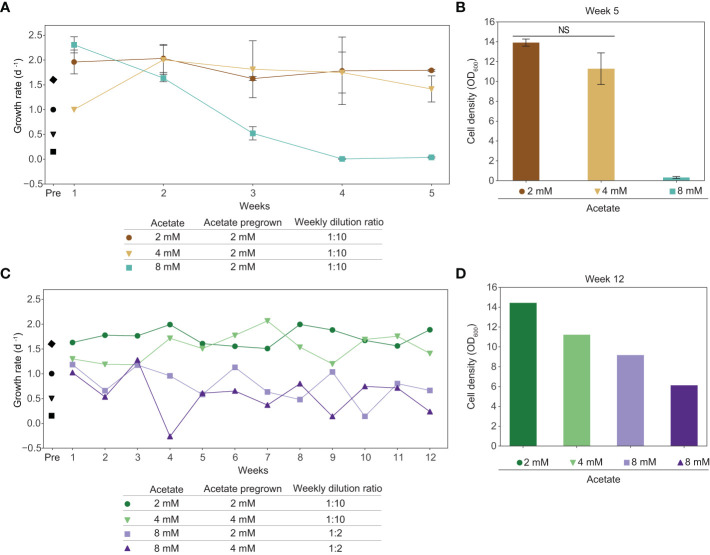
Adaptive laboratory evolution experiments of tobacco cell cultures result in increased acetate tolerance. **(A, B)** Cells were grown in media containing sucrose plus 2 mM acetate for 5 months prior to this experiment to allow for acclimation to acetate. At the initiation of this experiment, the culture was split into media containing sucrose plus 4 mM acetate, sucrose plus 8 mM acetate, or remained in sucrose plus 2 mM acetate. Cells were subcultured weekly at a 1:10 dilution ratio. Data points represent average between biological replicates (N=2). Error bars represent standard deviation. “Pre” indicates growth rate before adaptive laboratory evolution experiments, demonstrating the baseline growth rate. Black markers represent average growth rate of cultures grown prior to adaptive laboratory experiments, 3 biological replicates, one week of growth. This is the same data as shown in **Figure 3A**. Diamond: 88 mM sucrose + 0 mM acetate; circle: 88 mM sucrose + 2 mM acetate; triangle: 88 mM sucrose + 4 mM acetate; square: 88 mM sucrose + 8 mM acetate. **(A)** Growth rate based on OD_600_ measurements at the beginning and end of the week. **(B)** Cell density (OD_600_) on the last day of the experiment, at the end of the 5th week of growth. A two-tailed unpaired t-test was used to compare growth in media with sucrose plus 2 mM acetate to growth in media with sucrose plus 4 mM acetate, *p*-value is 0.149. NS denotes no significant difference from 2 mM (*p*-value > 0.05) **(C, D)** Cells were grown in media containing sucrose plus 2 mM acetate for 7 months prior to this experiment to allow for acclimation to acetate. At the initiation of this experiment, the culture was split into media containing sucrose plus 8 mM acetate or remained in sucrose plus 2 mM acetate. Additionally, cells were grown in media containing sucrose plus 4 mM acetate for 2 months prior to this experiment to allow for acclimation to acetate. At initiation, the culture was split into media containing sucrose plus 8 mM acetate or remained in sucrose plus 4 mM acetate. Cells grown in media containing sucrose plus 2 or 4 mM acetate were subcultured weekly at a 1:10 dilution ratio. Cells grown in media containing sucrose plus 8 mM acetate were subcultured weekly at a 1:2 dilution ratio. **(C)** Growth rate based on OD_600_ measurements at the beginning and end of the week. **(D)** Cell density (OD_600_) on the last day of experiment, at the end of the 12th week of growth.

We determined that exogenous acetate is metabolized by tobacco plant cell cultures, however, acetate is inhibitory at the concentrations likely needed to support substantial cell growth. The first step towards improving acetate utilization in tobacco plant cell culture is to improve acetate tolerance. We conducted an adaptive laboratory evolution experiment to increase the tolerance of plant cell cultures to higher concentrations of acetate by growing cells in media with sucrose plus acetate. We found that tobacco plant cell cultures can stably grow long-term in media with sucrose plus 2 mM acetate. We grew cells in this media for five months, approximately 80 generations, subculturing weekly at a 1:10 dilution ratio. These cells were then moved into media with higher concentrations of acetate and subcultured weekly at a 1:10 dilution ratio for a 5-week-long experiment (**Figures 6A, B**). Cells pregrown in sucrose plus 2 mM acetate were moved to media containing sucrose plus 4 mM acetate, in which they grew proficiently for the duration of the 5 week experiment. However, cells pregrown in sucrose plus 2 mM acetate that were moved into sucrose plus 8 mM acetate media started decreasing in growth rate immediately and fell to a growth rate below 0.1 d^-1^ by week 4.

In a second adaptive laboratory evolution experiment to improve acetate tolerance, we found that tobacco plant cell cultures can stably grow long-term in media with sucrose plus 2 or 4 mM acetate. Before the experiment began, we grew cells with sucrose plus 2 mM acetate for seven months, approximately 112 generations, and with sucrose plus 4 mM acetate for two months, approximately 32 generations, subculturing weekly at a 1:10 dilution ratio. Cultures pregrown in sucrose plus 2 or 4 mM acetate were moved into media containing sucrose plus 8 mM acetate and subcultured weekly at a 1:2 dilution ratio. We continued to subculture cells grown in sucrose plus 2 or 4 mM acetate at a weekly dilution ratio of 1:10. After 12 weeks, cultures in media containing sucrose plus 8 mM acetate were still growing but they had lower cell densities than cultures kept in sucrose plus 2 or 4 mM acetate. Additionally, cultures grown in sucrose plus 8 mM acetate in this second adaptive laboratory evolution experiment ended the twelve-week-long experiment with ODs above 5.0, a substantial increase over the final ODs for cultures grown in sucrose plus 8 mM acetate in the first adaptive laboratory evolution experiment, which were below 0.5 after just five weeks (**Figures 6C, D**).

## 3 Discussion

These experiments have advanced our understanding of the relationship between acetate and tobacco plant cell cultures, including growth effect, metabolism, gene expression, and potential for utilization as a primary carbon and energy source. By growing tobacco plant cell cultures with sucrose and varying amounts of acetate, we have defined the effect on growth for a range of concentrations. Below 1 mM acetate does not show a significant effect on dry weight, 2 to 4 mM acetate negatively affects cell growth, and above 8 mM acetate completely prohibits growth (**Figures 3A–C**). Our findings agree with the range of acetate concentrations found to inhibit growth of whole plants (1 to 3.5 mM) (Turner et al., 2005; Fu et al., 2020; Hann et al., 2022) and plant cell cultures (above 0.2 mM to above 10 mM) (**Table 2**). We further clarified these observations from published literature in which growth inhibition of plant cell cultures by acetate was not the primary focus of the study and systematically defined the effect of different concentrations of acetate on the growth of tobacco plant cell cultures.

To determine if acetate could support growth as the sole carbon and energy source, cells were grown in media containing acetate concentrations lower than the inhibitory threshold. We observed that 3.7 kJ l^-1^, from sucrose or acetate, does not contain enough energy to support substantial growth of tobacco plant cell cultures (**Figures 3D–F**). However in media with 4 mM or 2 mM acetate, a slight increase in dry weight is observed compared to cells grown with no carbon or energy source (**Figure 3E**, *p*-values 0.07 and 0.06; **Figure 4C**, *p*-value 0.0002), which suggests that these low concentrations of acetate support very minimal growth of tobacco plant cell culture.

Using [2-^13^C] acetate, we demonstrated that exogenous acetate is metabolized by tobacco plant cell cultures. ^13^C was incorporated into citrate/isocitrate, succinate, and malate which signifies that exogenous acetate is converted to biologically active acetyl-CoA and goes through the tricarboxylic acid (TCA) cycle to create energy (GTP, NADH, and FADH_2_) (**Figure 2**). ^13^C-labeled amino acids indicate that the cells can use exogenous acetate to build proteins. The labeled products and intermediates of gluconeogenesis demonstrate that exogenous acetate can also be used to build carbohydrates. Succinate and pentose stand out as the metabolites with the most extreme and consistent ^13^C enrichment among biological replicates of cultures grown with [2-^13^C] acetate (without sucrose). Our findings complement the few published studies looking at acetate metabolism in other species of plant cell cultures (**Table 1**). Carbon labeling from exogenous acetate in amino acids and TCA intermediates has been reported in plant cell cultures of Paul’s Scarlet rose (Fletcher and Beevers, 1970), maize (*Zea mays) (* Ashworth et al, 1987 *)*, and species of the Solanaceae family (Marty et al., 1997). However, previous descriptions of carbon incorporation from acetate into metabolites associated with gluconeogenesis in plant cell cultures are less common, but this work clarifies that this metabolism is possible.

There is a large discrepancy in ^13^C enrichment between cells grown with and without the addition of sucrose to media containing [2-^13^C] acetate. The most likely explanation for this difference is the dilution of ^13^C. The many metabolites made with unlabeled carbon from sucrose, which was present in a much higher concentration, may have diluted out the few metabolites made with labeled carbon from acetate. Although there is notably more ^13^C enrichment in cells grown with acetate alone compared to cells grown with acetate plus sucrose, we see enrichment in both conditions compared to cells grown without labeled [2-^13^C] acetate. An alternative explanation for this difference in labeling that would require further analysis to confirm, is catabolite repression, a process in which genes involved in the metabolism of alternative carbon sources are downregulated in the presence of the preferred carbon source (Gancedo, 1992; Graham et al, 1994; Lee et al, 1999; Beeckmans, 2001).

To explain why exogenous acetate is metabolized but cannot serve as the sole carbon and energy source, we explored expression of genes encoding enzymes involved in the glyoxylate cycle. Acetate is utilized as a carbon and energy source during early post-germinative growth of whole plants, when seeds are growing from storage lipids converted to acetate and used for growth via the glyoxylate cycle. Tissue-specific gene expression databases show that in *Arabidopsis thaliana* and *Nicotiana attenuata* (wild tobacco), genes encoding glyoxylate specific enzymes (MLS and ICL) are expressed primarily in seeds, with little expression in other parts of the plant (Brockmöller, 2015; Klepikova et al., 2016), demonstrating acetate is not well utilized in adult whole plants (Hann et al., 2022). Since we observed only minimal growth of tobacco plant cell cultures on acetate as the sole carbon and energy source (**Figures 3E**, **4C**), we hypothesized that tobacco plant cell cultures may also have low expression of glyoxylate cycle specific genes. Our analysis of the BY-2 transcriptome (Yang et al., 2015; Yang et al., 2017) supports this hypothesis. Genes encoding glyoxylate cycle specific enzymes are expressed at very low levels, while genes encoding enzymes from the TCA cycle and glycolysis are expressed at high levels. Previous studies have shown that growth conditions can alter expression of genes encoding glyoxylate specific enzymes (Kudielka and Theimer, 1983; Graham et al, 1994; McLaughlin and Smith, 1994; Lee and Lee, 1996; Ismail et al., 1997), which suggests these may be good targets for strain engineering to improve growth in acetate containing media. Increasing expression of glyoxylate cycle genes may be an important step towards improving growth of tobacco plant cell cultures on exogenous acetate, but first the cells must be able to tolerate acetate.

We cultivated our tobacco cell cultures in sub-inhibitory concentrations of acetate in order to evolve increased acetate tolerance in our strains. Our second adaptive laboratory evolution experiment was successful and allowed for tobacco cell culture growth in media with acetate concentrations that were previously inhibitory (8 mM). Between the two experiments, additional generations of growth in higher concentrations of acetate prior to the experiment as well as increased dilution ratios during the experiment may all have contributed to the improved growth of tobacco plant cell culture in media containing sucrose plus 8 mM acetate (**Figure 6**). Adaptive laboratory evolution experiments have successfully isolated microbial strains with increased acetate tolerance or utilization, including *E. coli (* Treves et al, 1998 *)*, *Saccharomyces cerevisiae (* Gilbert et al, 2009 *)*, and *Moorella thermoacetica* (an anaerobic bacterium) (Reed et al., 1987). Adaptive laboratory evolution experiments in plant cell cultures may take more time compared to other microbes due to their slower growth rate, but our experiments suggest that this approach could lead to cells more tolerant to higher acetate concentrations. Further efforts will be needed in order to reach acetate levels that are energetically comparable to more commonly used carbon substrates, like sucrose.

## 4 Conclusion

Acetate is an attractive alternative carbon source for the cultivation of plant cell cultures. We have made advances to better understand the current relationship between acetate and tobacco plant cell cultures and identified the next steps towards modifying plant cell cultures for robust growth with acetate. We established that acetate negatively affects tobacco plant cell culture growth even at concentrations as low as 2 mM, and above 8 mM inhibits growth completely. Cells grown in media containing acetate concentrations below the growth prohibiting threshold and no sucrose show an increase in dry weight compared to cells grown without sucrose or acetate, but these concentrations do not contain enough energy to support substantial cell growth. Our metabolomics study using [2-^13^C] acetate shows that tobacco plant cell cultures can metabolize exogenous acetate into metabolites involved in the TCA cycle, gluconeogenesis, and amino acid biosynthesis. We discovered that genes encoding enzymes from the glyoxylate cycle are expressed at very low levels compared to genes from the TCA cycle and glycolysis, which helps explain why cells are able to metabolize exogenous acetate but not utilize it for substantial growth. We used adaptive laboratory evolution experiments to successfully generate a more acetate tolerant tobacco plant cell culture; these cells can be used in future experiments for additive improvement of acetate tolerance and utilization. If large improvements can be achieved, acetate has the potential to replace or supplement sucrose in plant cell culture media.

## 5 Materials and methods

### 5.1 Plant cell culture growth

#### 5.1.1 Culture conditions

Tobacco BY-2 cell culture (*Nicotiana tabacum* cv. bright yellow 2) with tubulin tagged with RFP was used for all experiments. This strain was created by Dr. Takashi Hotta with the same pBIN41 pUBI10::mCherry-TUB6 construct as used in *Arabidopsis* in Fujita et al., 2013 (Fujita et al., 2013). Cells were grown in media containing 4.3 g MS (Murashige and Skoog basal medium powder) (Murashige and Skoog, 1962), 30 g sucrose, 100 mg Myo-inositol, 1 mg thiamine, 200 mg 2,4-Dichlorophenoxy acetic acid sodium salt monohydrate (2,4-D), and 200 mg KH_2_PO_4_ per liter as described at RIKEN BRC through the National BioResource Project of the MEXT, Japan (Experimental Plant Division, RIKEN BRC, 2014). When sucrose was included in the growth media it was at this concentration (30 g l^-1^, approximately 88 mM), unless otherwise noted. Acetic acid was added to media in addition or in place of sucrose to reach desired molar concentrations, unless otherwise noted. Potassium hydroxide was used to adjust the pH of the media to 5.8. 50 ml cell cultures were grown in 250 ml glass shake flasks with tops that allow gas exchange (Fisher 50-121-5154) covered in tinfoil to block light, at 20 °C (**Figures 3A–C**) or 22 °C (**Figures 3D–F**, **4**, **6**) on a shaking platform. Cells were subcultured weekly under aseptic conditions at a dilution ratio of 1:10 unless specified otherwise.

#### 5.1.2 Assays

For growth measurements, optical density (OD) measurements were taken at wavelength 600 nm using a QuickDrop Spectrophotometer (Molecular Devices) (Sakato and Misawa, 1974; Tanaka et al, 1992; Sparkes et al., 2006). Cells were well agitated immediately before measurements. Growth rates were calculated as change in OD per day (Hall et al., 2014). For dry cell weights the culture was centrifuged, washed with deionized water to remove residual salts, dried overnight at 80°C, and then weighed. In some instances, the culture was filtered through a Whatman filter paper rather than spinning down prior to drying (**Figures 3D**, **4C**). Photographs of flasks were taken using a Nikon 7500 DSLR camera.

#### 5.1.3 Statistical analysis

Two-tailed unpaired t-tests were used to compare experimental samples to controls. More details, including the number of replicates used, can be found in figure legends.

### 5.2 Metabolomics

#### 5.2.1 Sample preparation

[2-^13^C] acetate (Sigma Aldrich) was used for labeling in metabolomics assay. Cultures were started at approximately 1.0 OD_600_ and grown for two weeks. Samples were kept in -80°C until the analysis was performed. Samples were freeze dried, weighed (approximately 10 mg), and homogenized using a bead mill (3 2.8 mm beads per 2 ml tube). 1:2 water:methanol (v:v) was added (750 µl per sample), samples were vortexed for 60 minutes (4°C), chloroform was added (500 µl per sample), samples were vortexed again for 15 minutes (still at 4°C), and finally samples were centrifuged (10 minutes, 16,000 x g at 4°C) to separate the polar and nonpolar fractions. The polar fraction was analyzed by LC-MS.

#### 5.2.2 LC-MS

LC-MS metabolomics analysis was performed by the Institute of Integrative Genome Biology Metabolomics Core Facility at University of California, Riverside (UCR) using a Synapt G2-Si quadrupole time-of-flight mass spectrometer (Waters). Metabolite separations were carried out on an I-class UPLC system (Waters) using a ZIC-pHILIC column (2.1 x 150 mm, 5 µM) (EMD Millipore). Two mobile phases were used (A) water with 15 mM ammonium bicarbonate adjusted to pH 9.6 with ammonium hydroxide and (B) acetonitrile. The column was held at 20°C with a flow rate of 150 µl minute^-1^ and 2 µl was used as the sample injection volume. The following gradient was used: 0 min, 10% A, 90% B; 1.5 min, 10% A, 90% B; 16 min, 80% A, 20% B; 29 min, 80% A, 20% B; 31 min, 10% A, 90% B; 32 min, 10% A, 90% B.

The MS was operated in negative ion mode (50 to 1200 *m/z*) with a 100 ms scan time. MS/MS was acquired in data dependent fashion. The source temperature was 150°C and the desolvation temperature was 600°C. The desolvation gas was nitrogen (1100 l hr^-1^), the cone gas was also nitrogen (150 l hr^-1^), and the collision gas was argon. The capillary voltage used was 2 kV. Leucine enkephalin was infused and used for mass correction.

#### 5.2.3 Data processing and analysis

The open-source Skyline software was used for data processing (MacLean et al., 2010). Metabolites were identified by MS (less than 5 ppm) and MS/MS using the Metlin database (Guijas et al., 2018). Data for isocitrate and citrate are included as cumulative values (iso/citrate) because they were indistinguishable. Log_2_(^13^C enrichment) was calculated by:


$${log}_{2}(\frac{treatment(\frac{M+X}{M})}{control(\frac{M+X}{M})})$$

where M is the area under the curve of molecules made up of only ^12^C-isotope atoms, and M+X is the area under the curve of molecules with ^13^C-isotope atoms incorporated into the molecule, with X being the number of ^13^C-isotope atoms incorporated. Untreated replicates are a single replicate normalized to the average of all replicates, to better visualize the variation between control replicates.

### 5.3 Gene expression analysis

RNA-seq data was obtained from NIH accession number SRA091805 (Yang et al., 2015; Yang et al., 2017). Two transcriptomes, with and without methyl jasmonate treatment, were used as biological replicates. Reads were aligned to the *Nicotiana tabacum* reference transcriptome (Edwards et al., 2017). The genome was indexed using samtools (version 1.16) (Li et al., 2009). Bwa-mem2 (version 2.2.1) was used to generate read alignments with cDNA provided by Edwards et al, 2017 (Vasimuddin et al., 2019). Transcripts of the same functional annotation were summed to achieve TPM values used.

## Author contributions

ECH, JEM, and REJ contributed to the conception of this manuscript. ECH and REJ designed the acetate studies and ECH performed all experiments. MH-D. helped with metabolomics analysis and acetate metabolism in plant literature. AJG and ECH performed RNA-seq analysis. All authors contributed to the article and approved the submitted version.
